# Development and clinical implementation of a digital system for risk assessments for radiation therapy

**DOI:** 10.1016/j.zemedi.2023.08.003

**Published:** 2023-09-02

**Authors:** Dominik Kornek, David Menichelli, Jörg Leske, Michael Hofmann, David Antkiewicz, Tobias Brandt, Oliver J. Ott, Michael Lotter, Marga Lang-Welzenbach, Rainer Fietkau, Christoph Bert

**Affiliations:** a Department of Radiation Oncology, Universitätsklinikum Erlangen, Friedrich-Alexander-University Erlangen-Nürnberg (FAU), 91054 Erlangen, Germany; b Comprehensive Cancer Center Erlangen-EMN (CCC ER-EMN), 91054 Erlangen, Germany; c IBA Dosimetry GmbH, 90592 Schwarzenbruck, Germany

**Keywords:** Risk analysis, Failure modes and effects analysis (FMEA), Fault tree analysis (FTA), Risk-based quality management, Process throughput, Radiation therapy

## Abstract

Before introducing new treatment techniques, an investigation of hazards due to unintentional radiation exposures is a reasonable activity for proactively increasing patient safety. As dedicated software is scarce, we developed a tool for risk assessment to design a quality management program based on best practice methods, i.e., process mapping, failure modes and effects analysis and fault tree analysis. Implemented as a web database application, a single dataset was used to describe the treatment process and its failure modes. The design of the system and dataset allowed failure modes to be represented both visually as fault trees and in a tabular form. Following the commissioning of the software for our department, previously conducted risk assessments were migrated to the new system after being fully re-assessed which revealed a shift in risk priorities. Furthermore, a weighting factor was investigated to bring risk levels of the migrated assessments into perspective. The compensation did not affect high priorities but did re-prioritize in the midrange of the ranking. We conclude that the tool is suitable to conduct multiple risk assessments and concomitantly keep track of the overall quality management activities.

## 1 Introduction

Prospective risk analyses sensitize facilities to unsafe conditions and possible ways of failure of treatment processes before they are clinically implemented. As a method of choice, many practitioners utilized the (process) failure modes and effects analysis, (P-)FMEA, which systematically identifies potential failure chains within a defined process (see, e.g., references [1], [2], [3], [4], [5], [6], [7], [8], [9], [10], [11]). According to a recent German survey conducted by Baehr et al. [12], FMEA was the most common method applied by 52% of the 48 participating institutions. The FMEA usually includes a criticality analysis in which reports or experts are consulted to quantitatively evaluate these failure chains. Based on this evaluation, a surrogate for the criticality (e.g., risk priority number, RPN) is then calculated to precisely define the ranking for further actions. The prevalence of FMEA may be explained by larger organizations mainly demonstrating FMEA examples. In 2008, the World Health Organization published their Radiotherapy Risk Profile [13] with a list of 33 risks within a general process of care. Fundamentally, this list can be viewed as a simplified PFMEA. In 2009, the French Nuclear Safety Authority based their methodology for risk self-assessment inter alia on FMEA, Hazard Analysis Critical Control Point (HACCP) and the 5 M method (Machinery, Manpower, Material, Measurement and Method) and then decided to use FMEA due to easy implementation and prioritization of actions [14].

FMEA is advantageous if one wants to identify a high number of singular failure modes, i.e., failure modes immediately causing process failures. However, the method is not capable of modelling dynamics between failure modes even though it is known that failure modes indeed follow error pathways before they affect treatment negatively [15]. In their report N° 181 [16], the European Commission stated that fault tree analysis, FTA, is a suitable method for more in-depth assessments. In industrial engineering, FTA is used for functional modelling and reliability analysis [17]. It overcomes the aforementioned problem and could be input data previously obtained through FMEA. Then, in 2016, the AAPM TG-100 report demonstrated in great detail how both FMEA and FTA could be used in combination to better describe the risk profile of a treatment process [1].

Spreadsheets are commonly utilized to list the treatment process and to subsequently perform FMEA. However, visual tools such as flowcharts and fault trees are usually not supported. In consequence, several software tools are needed to describe the relationships between process steps, decision paths, failure modes and their dependencies as well as measures. These relationships should desirably be described by only a single dataset as the use of several software tools increases efforts to maintain data integrity. In addition, a potentially emerging problem is the overarching prioritization of failure modes from different individual risk assessments, e.g., concerning different treatment modalities of a clinic. After all, the total hospital resources are limited, and all required actions are drawn from these resources. In our specific case, two risk analyses concerning external beam radiation therapy (EBRT) in general and specifically with a Halcyon treatment unit (Varian Medical Systems Inc., Palo Alto, CA, U.S.) had previously been conducted. The question arises what the overarching ranking would be if all failure modes were compiled in a single combined list. To the best of the authors’ knowledge, there is currently no system commercially available to radiotherapy that offers solutions to the aforementioned problems.

Hence, the objectives of this study were as follows: First, a software that enables a formalized approach to risk assessment and combines FMEA and FTA was developed. Second, the tool was commissioned for clinical use and validated by means of existing risk assessments. Finally, a proposed method [18] was tested that gives different weights to the criticality (the RPNs) of failure modes from different workflows in order to calculate the overarching ranking.

## 2 Methods and materials

### 2.1 Software development

The tool, myQA® PROactive, was developed as a web database application managed by the SQLite database engine (public domain) and served by a Kestrel server within an ASP.NET Core framework (.NET Foundation, Redmond, WA, U.S.). Embedded into the network of the department, facility members could access the web interface with any available workstation since no client-wise installation was required. Three complementary risk assessment tools were implemented: FMEA, FTA, and Failure Modes and Effects Summary (FMES) as detailed in the upcoming sections.

#### 2.1.1 FMEA

FMEA is a systematic method that identifies potential failure chains whereby a failure chain consists of a failure cause, failure mode and failure effect [19]. Failure modes are manners in which process failures occur. Traditionally, a ‘bottom-up’ approach is followed, i.e., processes at a detailed level are scrutinized one after another and possible consequences of failures on higher levels are deduced in a subsequent step. Alternatively, a ‘top-down’ approach may be applied where first process functions are identified and then those functions that could contribute to pre-identified top events are investigated [20]. After the failure analysis, failure chains are quantitatively evaluated in order to indicate the criticality. During this criticality analysis, the occurrence *O* of the failure causes, the detection *D* of the failure causes or the failure modes as well as the severity *S* of their respective effects are estimated. Finally, barriers are identified to optimize the process, beginning with the highest rated failure modes.

To facilitate an industry level design, the technical norm IEC 60812 [20] was consulted to implement FMEA. More specifically, a dedicated user management was implemented to enable specific functions within a team, i.e. roles for administrators, moderators and analysists. The moderator role was given all privileges including version control and deleting risk analyses while analysists were limited to only edit risk analyses. Furthermore, it was decided to use the RPN as a criticality surrogate. The RPN is the product of *S*, *O*, and *D*. These three parameters were implemented as presented in the TG-100 task report (see Table II in [1]), i.e., as a ten-step rating system with scores ranging between 1 and 10. Moreover, barriers were characterized to be either proactive or reactive. Whereas proactive barriers aim to prevent the failure mode, reactive barriers are supposed to detect the failures in case they did occur. In other words, proactive barriers reduce *O* and reactive barriers increase *D*. This mechanism was achieved by introducing a reduction factor $P_{\mathrm{miss}}$ that estimates the effectiveness of the respective barrier. By assuming barriers i to be independent of each other, the residual (optimized) RPN could be obtained with ${\mathrm{RPN}}^{\mathrm{out}}=S\cdot O\left(P_{\mathrm{occ}}^{\mathrm{in}}\cdot \prod_{i}P_{i,miss}^{\mathrm{proactive}}\right)\cdot D\left(P_{\det}^{\mathrm{in}}\cdot \prod_{i}P_{i,miss}^{\mathrm{reactive}}\right)$ (see Supplementary Materials for an example).

#### 2.1.2 FTA

FTA is a ‘top-down’ Boolean logic tool describing quantitatively or qualitatively fault events building an error pathway through logical operations leading to a top event. A graphical tree is developed, whereby the top event is the pre-identified event of interest and positioned on the top of the fault tree. Starting from the top, its possible branches are developed further downstream to identify associated intermediate and basic events of failure that contribute to the top event. Events may be occurred failure modes or failed barriers. Basic events are such events not developed any further as they may be the failure causes or beyond the system boundaries. The design of the tree and the logical gates followed the technical norm IEC 61025 [17].

#### 2.1.3 Failure modes and effects summary (FMES)

FMES is a method for grouping failure modes of an FMEA that produce the same potential effect. It is performed in order to reduce the data input for higher level FMEAs or FTAs [21]. First, all failure modes are analyzed and their potential effects on the highest system level are categorized. Then, all failure modes in the FMEA that cause the same effect are summarized as one failure mode in the FMES, with the failure modes of the FMEA then becoming its causes.

**Figure 1:**
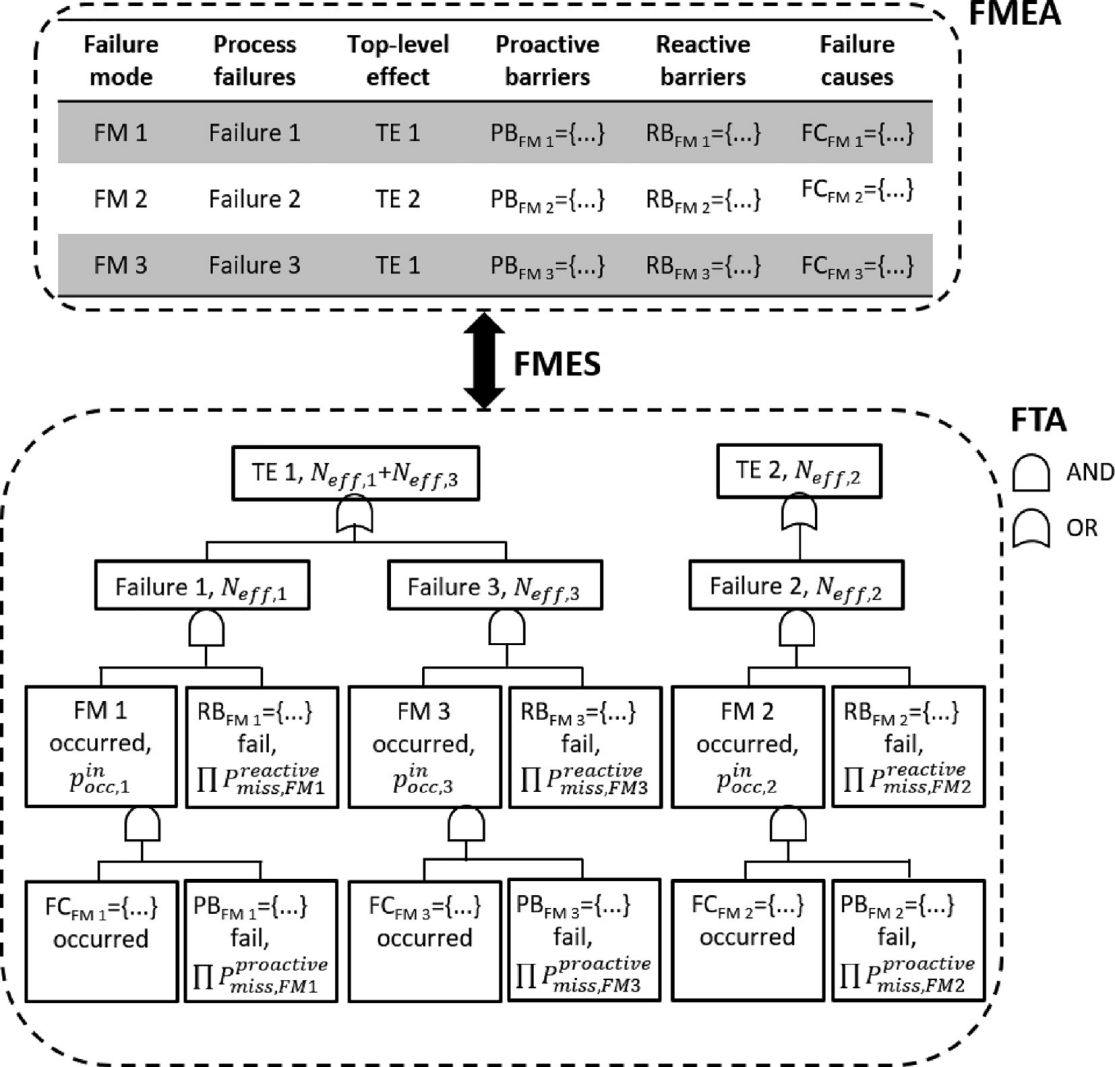
FMES: Failure modes and effects summary, FMEA: failure modes and effects analysis, FTA: fault tree analysis. FMES groups failure modes of the FMEA by the same effect on the highest level. All the failure modes within a cut set serve as input for fault events in the fault tree, with the effect on the highest level as the top event.

Risk assessment data were able to be converted between FMEA and FTA by applying the FMES algorithm as depicted in Fig. 1. Failure modes of the FMEA and the fault events of the FTA were treated the same to this end. This allowed for the results obtained by FMEA and FTA to be represented both as a table and as a fault tree. By combining both methods, the rate $N_{\mathrm{eff}}$ that a top event occurs and remains undetected could be computed. As can be seen in Fig. 1, each branch is described by a single failure caused by a failure mode/fault event and barriers are connected by AND gates. Therefore, $N_{\mathrm{eff},j}=P_{\mathrm{occ}}^{\mathrm{in}}\cdot \prod_{i}P_{i,miss}^{\mathrm{proactive}}\cdot P_{\det}^{\mathrm{in}}\cdot \prod_{i}P_{i,miss}^{\mathrm{reactive}}\cdot TP$ for each failure j and barrier i. TP was the absolute patient throughput per year. Using OR gates to join the branches, the respective event rates on the next higher level were then given by $\sum N_{\mathrm{eff},j}$. As an additional way for prioritizing, only branches exceeding a particular threshold for *S* could be displayed and those contributing higher shares to the top event further analyzed.

### 2.2 Software validation

Software validation answers the question whether a tool is suitable for performing the intended task (here: risk assessment). The tool was validated by commissioning followed by the migration of existing risk analyses as described in the following sections. Validation was considered successful as soon as performing and continuing the risk assessment was equally feasible as before.

#### 2.2.1 Commissioning the software

With the intent to use the ‘top-down’ FMEA approach for future risk assessments, the software was commissioned for clinical use according to the following steps.

Firstly, it was decided to use a standard process map valid for almost all future risk assessments. As Gilmore and Rowbottom [4] showed, they were able to assign their local process steps and failure modes for lung radiotherapy to a general EBRT process map provided by the AAPM [22]. This process map contained 91 process steps and was originally compiled to standardize incident reporting systems [22]. To reduce overlap between future EBRT risk assessments, this approach of using a standard process map was adopted here as well.

Secondly, functions and failures of the general EBRT process were established. The process functions were identified by analyzing the underlying purpose of each process step. By accurately and concisely describing the process functions, process failures could simply be deduced by negating these functions. This step was undertaken to establish the same wording for common events. Furthermore, using particular functions to create subsets of the process map was expected to help identifying more relevant failure modes.

Thirdly, the rating system was adjusted according to the established procedure of our department. Instead of rating the occurrence probabilities, frequencies as in failures per unit of time were the preferred choice. The severity parameter was also adjusted to our needs and associated with top events known from literature [1], [22], [23], [24].

Lastly, with the help of FMES the failure modes from two existing risk studies were analyzed in order to identify further top events (for a description of these studies see Section 2.2.2). Again, this was done to ensure consistent wording of failure effects. The top events were then used for FTA and placed on top of the process failures described above.

#### 2.2.2 Re-analyzing existing risk assessments

Prior to this work, two risk assessments had been performed by our department. The first study had identified 38 failure modes of our general EBRT workflow. Failure chains had been identified for the worst-case scenario using FMEA and evaluated using a 5x5 *S* vs. *O* risk matrix with three classes (green: ‘acceptable risk’, orange: ‘monitor risk, tolerable if ALARP^1^’ and red: ‘critical risk, measures required’) (see [25] for a detailed description of the methodology). In the same manner, a second risk study about the commissioning and operation of the Halcyon treatment unit comprising 19 failure modes had been performed (data not published).

In order to migrate these assessments to the commissioned tool, they had to be re-analyzed including a re-evaluation of the failure chains to be conformal to the more advanced methodology of the new software: The local process maps were assigned to the new standard process map. Failure effects were rephrased to be consistent with the process failures and top events. Barriers were classified as either proactive or reactive and given a probability of failure $P_{\mathrm{miss}}$ based on the ratio of the initial and residual occurrence rates. In a next step, the previous evaluations were converted to the new rating system as described in the previous section. The software did not support changing the rating system and thus, the rating criteria had to be manually mapped to their respective scores.

Ranks were assigned to the failure modes after sorting by descending RPN (‘rank by RPN^in^’). Failure modes sharing the same RPN also shared the same rank. Similarly, ranks were attributed to the original risk matrices by multiplying *O* with *S*. These products, known as risk scores [26], were then sorted descendingly. By means of the Spearman’s rank correlation coefficient *r_S_*, the following metrics were compared for plausibility: the initial risk matrix and RPN rankings, the initial *S/O* and re-evaluated *S/O* numbers as well as the mean RPN and mean risk score.

### 2.3 Combining risk assessment rankings

Janssens and van der Horst [18] suggested a method for making risk assessment results of different processes comparable. The method modifies the RPN by taking into account a fourth factor *T* that considers the throughput of process steps. More specifically, *T* is related inversely to the throughput. The following example illustrates the idea behind this concept: If a failure occurs once a month (*O* = 4) in a process with a high throughput of 200 patients per month compared to a lower throughput of 20 patients per month, then the relative performance in the latter case is much worse. Therefore, whenever the throughput is low, especially in cases of highly complex treatments, the resulting RPNs should be more emphasized. Here, scores for *T* were applied to the patient throughput ranging between less than ten and up to 5000 patients per year as shown in Table 2. The reader is referred to the original article [18] where the sundry effects of *T* are described in detail.

The re-analyzed failure modes were given a T score based on the patient throughput obtained by querying the oncology information system MOSAIQ (Elekta Inc., Sunnyvale, CA, U.S.). The total throughput of the general EBRT workflow was approx. 1600 patients per year of which approx. 19% were part of the Halcyon workflow. The modified RPNs were denoted as RPN* = RPN·T. Rankings obtained by both RPN and RPN* were compared. As the tool did not support RPN* evaluation at the time of this study, data exports were used for further processing on a separate spreadsheet.

## 3 Results

### 3.1 Software development

A dedicated risk assessment online tool for radiotherapy applying a combined FMEA-FTA approach was developed. The workflow was designed to systematically guide the user through all steps of the risk assessment. The first step of the workflow required the user to create the risk analysis under selection of a rating system. Subsequently, a process map was required which was described in the tool by a table or by a flowchart. At this point, the actual failure and risk analysis could be carried out. The creation of a failure mode always required the specification of a failure cause and effect as well as a rating of the parameters *S*, *O*, and *D*, resulting in an initial RPN^in^. This value which refers to the worst-case scenario could be reduced by adding either proactive or reactive barriers, i.e., introducing a reduction factor $P_{\mathrm{miss}}$. After the creation of a failure mode, the tool automatically generated the equivalent fault tree representation by applying FMES (the failure cause became the basic event, the failure mode became the fault event and the failure effect became the top event as shown in Fig. 1). Moreover, all failure modes resulting in the same top event became the branches of a single fault tree. The workflow of the software and an exemplary fault tree consisting of two failure modes can be found in the Supplementary Materials.

### 3.2 Software validation

#### 3.2.1 Commissioning the software

myQA® PROactive was commissioned for clinical use with the intent of a combined FMEA-FTA approach in general and a ‘top-down’ FMEA approach in particular. To this end, the AAPM’s EBRT process map [22] was adopted and analyzed.

Based on this map, nine distinct process functions and 36 process failures could be identified (Table 1). Only failures potentially affecting irradiation of patients negatively were included. As no further risk assessment has been performed since the commission of the tool, the effectiveness of using process failures could not be tested in terms of identifying relevant failure modes. However, we expect them to make FMEA more efficient as discussed later on.

**Table 1:** Processes of external beam radiation therapy and corresponding process failures. Process functions are used in ‘top-down’ FMEA to identify specific failure modes compromising those functions [20]. Dx: diagnosis, Rx: prescription, Fx: fraction.

Process	Process functions	Process failures
Patient assessment [22]	Patient identification	Failure due to wrong patient
	Treatment design: Accurate assessment of medical history and conditions	Failure due to incorrect or missing reports or records
		Failure due to incorrect interpretation of medical information
	Treatment design: Informed treatment decision	Failure due to wrong treatment decision
Imaging for RT planning [22]	Patient identification	Failure due to wrong patient
	Reproducibility: Manufacturing of immobilization devices	Failure due to faulty manufacturing (materials, durability etc.)
	Reproducibility, localization: Reproducible positioning and immobilization of patient for optimal target localization and organ sparing	Failure due to wrong patient positioning or immobilization
		Failure due to irreproducibility or infeasibility
		Failure due to wrong anatomical area
	Localization: Image acquisition with adequate spatial resolution and field-of-view	Failure due to poor image quality or inadequate field-of-view
Treatment planning [22]	Patient identification	Failure due to wrong patient
	Localization: Correct registering of other imaging studies with primary CT	Failure due to incorrect or missing imaging studies
		Failure due to incorrect interpretation
		Failure due to incorrect registering
	Localization, Optimization: Delineation and contouring of targets and organs-at-risk with exact geometrical precision	Failure due to wrong localization
		Failure due to contouring errors
		Failure due to lack of dose specifications or constraints
	Optimization: Calculation of optimal and deliverable treatment plan in congruence with Rx	Failure due to poor planning execution
		Failure due to plan calculation error
		Failure due to inaccurate beam model
Pre-treatment review and verification [22]	Validation: Approval of correct Dx, Rx and correctly scheduled Fx	Failure due to poor review/approval of poor plan
		Failure due to wrong scheduling
	Validation: Approval of verified treatment plan	Failure due to erroneous check or measurement (execution, evaluation etc.)
Treatment delivery [22]	Patient identification	Failure due to wrong patient
	Treatment delivery, reproducibility, localization: Delivery of treatment plan according to simulation	Failure due to machine errors
		Failure due to deviations from simulation
On-treatment quality management [22]	Validation: Review of treatment progress	Failure due to inadequate evaluation
Post-treatment completion [22]	Patient identification	Failure due to wrong patient
	Administration: Referral to other attending physicians	Failure due to organizational issues
	Validation: Review of treatment success	Failure due to inadequate monitoring strategies
General	Administration: Management of coherent process sequence	Failure due to communication/documentation errors
		Failure due to data transfer error
		Failure due to data entry errors
		Failure due to delay
		Failure due to identification errors
		Failure due to organizational issues

**Table 2:** Scores and corresponding meanings for severity *S*, occurrence *O*, detection *D* and process throughput *T* taken and modified from [1], [18], [20], [22], [23], [24].

Score	Severity *S*		Occurrence *O*	Detection *D*	Throughput *T*
	Top event	Medical harm			Patients per year
1	No effect	No harm	Less than once a year	Almost certain	5000
2	Inconvenience	Inconvenience (∼ minutes)	Once a year	Very high	2500
3		Inconvenience (∼ hours)	Several times a year (∼3)	High	1250
4	Suboptimal treatment	Side effects (no intervention)	Once a month	Moderately high	640
5	Unexpected deterministic side effects, wrong dose, wrong dose distribution, wrong location, wrong treatment period, wrong volume	Side effects (intervention)	Several times a month (∼3)	Moderate	320
6		Mild toxicity or tumor underdosage	Once a week	Low	160
7		Moderate toxicity or tumor underdosage	Several times a week (∼3)	Very low	80
8		Severe toxicity or tumor underdosage	Each day	Remote	40
9		Life-threatening	Several times a day (∼3)	Very remote	20
10		Premature death	Many times a day	Almost impossible	10

The parameters of the rating system were adjusted to our institution-specific needs and are given in Table 2 and in full detail in the Supplementary Materials.

Using FMES, the highest-level failure effects of the 57 failure modes could essentially be summarized in 11 top events given in Table 3. A many-to-many relationship existed between the process failures and top events, i.e., a particular process failure can result in more than one top event.

**Table 3:** Top events identified in both the risk assessments for ^a)^ the general EBRT workflow and ^b)^ for percutaneous treatments using specifically the Halcyon. Arithmetic means are shown for the evaluated parameters of the original and re-analyzed assessments. The number of identified failure modes for each top event is indicated in the brackets. S: severity, O: occurrence, D: detection, S x O: risk score, RPN: risk priority number.

		Risk matrix			Risk priority number			
Top event (Count)	Description	$\overset{¯}{S}$	$\overset{¯}{O}$	$\overset{¯}{S\times O}$	$\overset{¯}{S}$	$\overset{¯}{O}$	$\overset{¯}{D}$	$\overset{¯}{\mathrm{RPN}}$
TE 1 (4): Unexpected deterministic side effects ^a)^	Deterministic side effects that were not expected for the planned treatment	2.50	4.75	11.88	7.00	6.00	4.25	178.50
TE 2 (1): Wrong treatment period ^a)^	Correct absolute dose but incorrect fraction pattern, e.g., 10 instead of 25 fractions	5.00	5.00	25.00	9.00	8.00	2.00	144.00
TE 3 (3): Non-radiation induced harm ^a)^	Bodily harm, e.g., due to contrast allergy, gantry collision etc.	3.00	3.70	11.10	5.67	3.67	6.67	138.80
TE 4 (10): Wrong absolute dose ^a), b)^	Incorrect total dose delivered	3.30	4.30	14.19	6.50	4.80	4.20	131.04
TE 5 (6): Wrong volume ^a), b)^	Correct anatomical site but incorrect volume treated, e.g., due to Treatment plan mix-up	2.50	4.30	10.75	5.00	5.00	4.17	104.25
TE 6 (6): Suboptimal treatment ^a)^	Any deviation from optimal treatment, e.g., suboptimal energy, unused bolus etc.	3.00	4.80	14.40	5.33	6.33	2.33	78.61
TE 7 (12): Inconvenience ^a), b)^	Minor problems causing delay or loss of comfort for patient or staff	2.25	4.80	10.80	3.00	5.83	3.58	62.61
TE 8 (1): Device defect ^b)^	Device defect causing downtime	1.00	3.00	3.00	3.00	3.00	5.00	45.00
TE 9 (2): Exposition of individuals not in treatment ^b)^	Unintentional irradiation of individuals other than patients, e.g., staff	2.50	2.00	5.00	7.00	2.00	2.50	35.00
TE 10 (1): Wrong dose distribution ^b)^	Correct absolute dose but incorrect dose distribution delivered, e.g., due to unplanned scatter material, leaf motion errors etc.	1.00	3.00	3.00	5.00	1.00	6.00	30.00
TE 11 (5): No effect ^a), b)^	Effect negligible	1.00	3.40	3.40	1.00	5.40	3.60	19.44

#### 3.2.2 Re-analyzing existing risk assessments

During a migration period of two weeks, the risk assessments were re-analyzed on a separate spreadsheet and then imported into the tool. During the process, six failure modes were merged with similar ones, resulting in 51 remaining failure modes. These were then associated with the process failures given in Table 1 and top events in Table 3. Four failure modes (e.g., ‘billing of fraction not performed’, TE 11: no effect) could not be assigned to a process failure. The ten highest ranked failure modes by means of the RPN are given in the Supplementary Materials.

**Figure 2:**
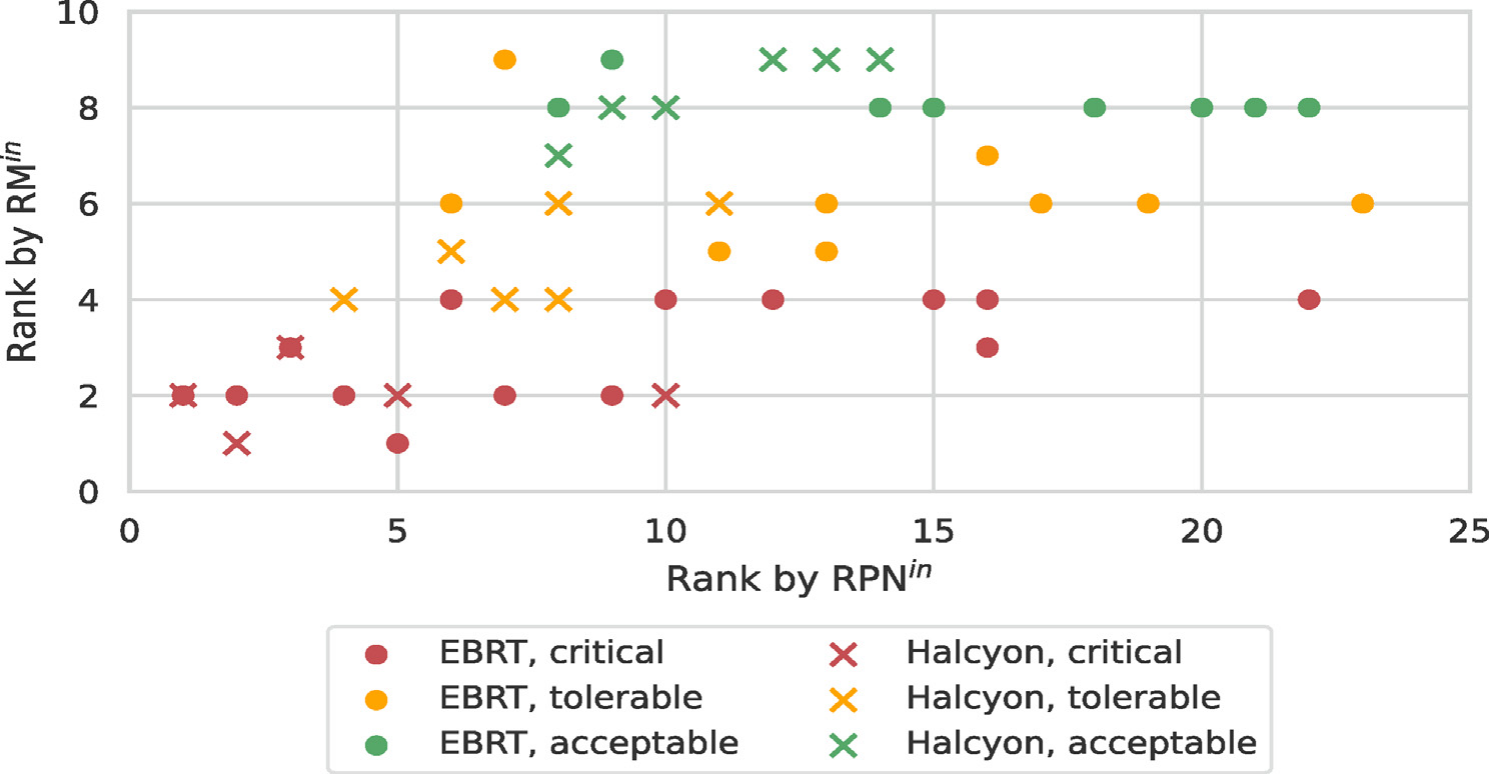
Comparison of the ‘prior’ ranking of failure modes of different treatment techniques obtained by the risk matrix and risk priority number. Both the EBRT ranking and Halcyon ranking are viewed independently, i.e., they both start at rank 1 using either RM or RPN as criticality. Instead of 51 only 48 data points can be seen due to overlap.

The initial ranks obtained by applying the RPN and risk matrix were compared for plausibility. A significant shift of ranks was introduced, however, a moderate to high degree of correlation could be maintained as can be seen in Fig. 2. The rank correlation coefficient was *r_S_* = 0.49 (*p* < 0.01) and *r_S_* = 0.81 (*p* ≪ 0.01) for the rankings of the EBRT and Halcyon assessment, respectively. For *S* and *O*, *r_S_* = 0.75 (*p* ≪ 0.01) (EBRT) and *r_S_* = 0.73 (*p* ≪ 0.01) (Halcyon) as well as *r_S_* = 0.60 (*p* ≪ 0.01) (EBRT) and *r_S_* = 0.74 (*p* ≪ 0.01) (Halcyon), respectively. As a result of using the RPN, failure modes that had been classified as acceptable or tolerable gained priority over failure modes that had previously been critical. Eight previously acceptable failure modes of the EBRT assessment had ranked between 8th and 9th. After re-evaluation, these eight failure modes ranked between 8th and 22th, taking up 65.2% of the entire range of obtained ranks which is nearly three times as much as before (22.2%). This is a result of increasing the informational content of evaluating because, with the risk matrix, 14 unique risk scores were obtainable (using a five-step scale) and with the RPN, 120 unique RPN scores (using a ten-step scale). Therefore, one risk score corresponded to 8.6 RPN scores on average. As an example, the failure mode ‘medical clarification of parameters affecting external imaging (e.g., MRI) not required’ was originally ranked fourth (*S* = 3, *O* = 5). With an RPN of 12 (*S* = 2, *O* = 6, *D* = 1), the new rank was only 22th. Furthermore, the criteria of the rating system changed. Whereas the maximum occurrence for the risk matrix was *O* = 5 meaning once a month or more often [25], the new rating system allowed to estimate the occurrence between once a month (*O* = 4) and up to many times a day (*O* = 10). This resulted in RPNs differing by a factor of up to 2.5. However, this increase in granularity was needed as 33 out of 51 (64.7%) failure modes had been rated *O* = 5 in the previous ranking. In contrast, 4% of failure modes were rated *O* = 10. In consequence, the mean priorities of the top events changed as well (see Table 3), yet the correlation was still high, with *r_S_* = 0.78 (*p* < 0.01).

It should be noted here that the software was designed with a fixed severity score applying the concept that regardless the likelihood of occurrence the severity will not change as long as the failure mode exists. However, it was found that certain types of safety interventions do decrease the severity. For instance, the barrier ‘structure set templates’ for the failure mode ‘OAR not delineated / contoured’ not only reduced the occurrence but also the severity score since the severity increases the more structures have not been contoured and could subsequently receive harm. Another example is the failure mode ‘inaccurate beam model in non-vendor TPS’. When accuracy is increased through more precise measurements, dose deviations and thus severity are decreased. In consequence, failure modes with initially high severity scores may demand more barriers than actually needed to be tolerable.

### 3.3 Combining risk assessment rankings

**Figure 3:**
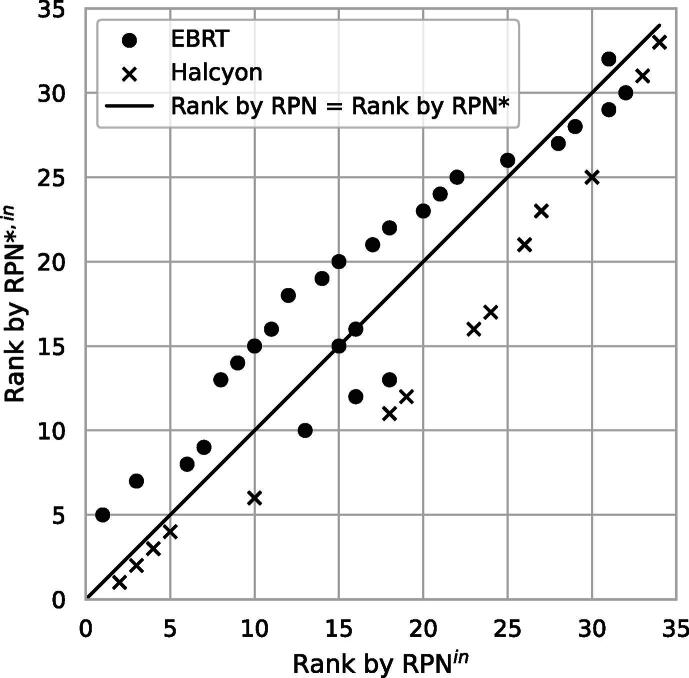
Effect of T on the overall ranking of failure modes of different treatment techniques and patient throughputs. Failure modes shifted above the line of origin are lowered in priority and vice versa. Failure modes on the line of origin kept the same priority. Instead of 51 only 41 data points can be seen due to overlap.

All RPNs were multiplied with a fourth parameter *T* to obtain the RPN*. Sorting descendingly by RPN* resulted in ranks being shifted as can be observed in Fig. 3 and the Supplementary Materials. The quintessential ranking did not change, i.e., high priorities stayed rather high and vice versa. However, there was a noticeable change in priorities in the mid-range. Failure modes of medium risk and higher throughput (EBRT) were effectively lowered in priority compared to failure modes of medium risk and lower throughput (Halcyon). Failure modes ranked first in the EBRT and Halcyon analyses were ‘target contour changes after plan approval not communicated (adaption to new medical findings)’ (RPN^in^ = 294) and ‘wrong placement of measuring instruments b)’ (RPN^in^ = 288), respectively. Even though the first had a higher initial RPN, it would position fifth in the combined ranking, shifted behind four Halcyon-only failure modes. As *T* ranged between 2 and 3 and between 5 and 6 for the EBRT and Halcyon failure modes, respectively, most Halcyon failure modes were essentially given twice the weight over EBRT failure modes. An entire comparison of these two assessments cannot be given here, rather it is the process of combining risk assessments in general that is emphasized.

## 4 Discussion

We developed and put into operation a user-friendly online system enabling a systematic and integrated FMEA-FTA workflow. The combination of both FMEA and FTA has been reported in several studies [27], [28], [29], [30] and were, in the field of radiotherapy, originally suggested by Huq et al. for evaluating quality assurance (QA) needs [31]. They were later included in the AAPM TG-100 report as means of designing a risk-based facility QA program [1]. Other task group reports such as AAPM TG-142 [32] recommended applying TG-100 (FMEA) as a means to support decisions when changing the QA program, e.g., when decreasing QA test frequencies. In contrast to optimizing QA programs, FMEA and FTA have also been recommended for assessing patient safety of entire treatment procedures [14], [16], [33], i.e., beyond the activities of the physicists’ QA program.

In our department, solely FMEA has been applied for assessing patient safety [25]. With the novel tool, we could replace remaining spreadsheets and introduce FTA into our assessments. After migrating existing risk assessments, error pathways could be visualized and better understood than before as foreseen in [34]. On the basis of FMEA alone, overall reliability cannot be deduced [35]. With fault trees complementing the FMEA, further quality assurance checks could be identified that increased the overall robustness of treatment procedures. However, it should be noted that a simplified FTA architecture was implemented, and as a result, fault trees could only depict an FMEA failure chain. Fault trees were therefore rather wide and shallow. A general FTA, however, usually identifies more levels. In three fault tree analyses reported for high-dose-rate brachytherapy, up to five levels of hierarchy were produced [36], [37], [38]. Therefore, fault trees converted from the FMEA should thus serve only as starting points for subsequent FTAs. The quality of both the FMEA and FTA may then be improved recursively [29].

In Table 1 potential process failures affecting patient safety are listed. We believe using the ‘top-down’ FMEA method and these listed failures will make future FMEAs more efficient. A problem of the traditional ‘bottom-up’ method is that any manner of failure represents a failure mode. When failure effects are only determined afterwards, then chances are failure modes are identified that cause no harm for the patient at all. In our case, this was, e.g., ‘billing of fraction not performed’ and four other failure modes. In the FMEA by Teo et al. about the commissioning process of their Halcyon, ‘water spilled over the edge of the tank’ was a failure mode [39]. This may be a failure mode indeed, however, we believe risk mitigating resources could be used more efficiently if irrelevant failure modes were not identified in the first place. With these process failures, our five failure modes having no effect would likely not have been identified as they could not be assigned to any of the listed process failures.

In Fig. 2, it can clearly be seen that re-evaluation led to significant changes in the ranking. The result showed that risk acceptance classes designed for the risk matrix could not be blindly applied to the RPN concept because failure modes that had been ‘acceptable’, ‘tolerable’ and ‘unacceptable’ before shared the same rank afterwards. On the one hand, subjectivity of re-evaluation contributed partly to this outcome. On the other hand, the number of rating steps and step sizes were increased. Of course, the more failure modes share the same rank, the less meaningful prioritized lists will be. Due to better differentiation of ratings and higher number of ranks with the RPN concept, the new ranking was accepted as more accurate. The problem of shifted priorities arose here because *S*, *O*, and *D* have been scored directly in terms of the available rating numbers. If, however, the underlying numerical values were estimated and the rating number only deduced afterwards (as exemplified in the Supplementary Materials), then risk evaluations could be more easily converted between rating systems. The software allows exactly that, entering a numerical probability which has a fixed relationship in any rating system.

If multiple risk analyses are conducted within a department, resources for measures should be managed efficiently by bringing all risk analyses into perspective. Sziklavari et al. reported that quality correlates with yearly patient volumes and minimum volumes must be achieved to fulfill standards [40]. Frequent techniques are likely to be of a very high quality already and, by implication, low volume processes may not. Therefore, having failure modes of processes of low throughput be more emphasized allows to mitigate potential risks to a small group of individuals earlier. The RPN*-method can be seen as an application of the equity principle that demands a minimum protection for all individuals [41]. On the other hand, *N_eff_* aims at the overall reduction in number of events. This is an application of the utility principle where limited resources are used to maximize the benefit, even if some individuals are left to be exposed to very high risks [41]. Both the RPN*- and the *N_eff_*-sorted lists may be considered to find an adequate trade-off between providing protection to all individuals as well as reducing the total number of events.

One potential shortcoming of the tool was the lack of ability to reduce the severity rating. In literature, there is no clear recommendation whether the severity score should be fixed or changeable. For instance, TG-100 literally states that ‘nothing will reduce the severity’ [1]. They argue that *O* and *D* scores can be reduced through control mechanisms, and high-risk failure modes should rather be eliminated or mitigated through process redesign which might introduce new failure modes in return. In contrast, the “Guide de l'ASN n°4” by the Autorité de sûreté nucléaire [14] and SEVRRA [42], a freeware tool for the evaluation of risk on radiotherapy, allow for the reduction of the severity. Both approaches have their advantages and disadvantages and further analysis is needed to determine a generally acceptable approach. Possible methods for implementing severity reducers in both proactive and reactive barriers will be investigated in future studies. In addition, the reduction factor $P_{\mathrm{miss}}$ of barriers was estimated by the risk management team. To allow for a consistent estimation and to consider the robustness of different types of barriers (e.g., an interlock is considered fail-safe whereas an SOP might fail due to poor human execution), a parametrization similar to occurrence and detection scores could be beneficial. SEVRRA is using a point system for rating the robustness of barriers [43]; in our tool, this approach would correspond to a probability look up table.

Another shortcoming is the reliance on the RPN concept itself. Even though the RPN is straightforward in its application which has contributed to its widespread popularity, including the present study, it also has numerous limitations. Lo and Liou summarized these limitations, among them, for example, the lack of consideration of the relative importance of *S*, *O*, and *D*, and the possibility of the same RPN being produced by different S-O-D combinations which, in turn, hide risk implications that may be entirely divergent [44]. As economic components such as budget limitations and process throughputs do not contribute to the RPN, the resulting ranks may also not be the most ideal order for implementing barriers. Buchgeister and Hummel made further points, inter alia the RPN’s lack of mathematical definition and oversensitivity toward mean values of *S*, *O*, and *D*, and stated that the RPN, in contrast to its name, is not suitable for risk prioritization, as larger RPNs do not also necessarily correspond to larger risks [45]. A promising remedy is the action priority, AP, a novel prioritization method introduced by the automotive industry [19] that overcomes many of the limitations of the RPN. The AP is a three-dimensional look-up table yielding the urgency of action for each individual combination of *S*, *O*, and *D*. In future studies, the AP should be investigated for a potential application in radiotherapy.

## 5 Conclusion

Radiation therapy is characterized by complex human-machine systems, extensive use of IT systems and constantly changing processes following new developments. TG-100 methodology allows for meaningful design of quality management programs. A digital system is required to keep track of the overall quality management program. Therefore, a dedicated software integrating all TG-100 tools was developed. The software was deemed suitable for performing risk assessments for radiotherapy. Long-term results of using the software following the successful data migration are subject of future studies.

## Declaration of Competing Interest

The authors declare the following financial interests/personal relationships which may be considered as potential competing interests: The inventors with the Department of Radiation Oncology, Universitätsklinikum Erlangen, and IBA Dosimetry GmbH have a patent pending on some functionality of myQA® PROactive.

## Footnotes

1 ALARP – as low as reasonably practicable
