# Supplementary material for: Development and clinical implementation of a digital system for risk assessments for radiation therapy
Source: Z Med Phys. 2023 Sep 2;34(3):371–83. doi: 10.1016/j.zemedi.2023.08.003 (PMC11384085; doi:10.1016/j.zemedi.2023.08.003)
Supplement: Supplementary data 1 [file mmc1.docx]

**Supplementary Materials**

# Terms and Definitions

| Criticality | Generic term for a ranking method (e. g., ‘RM’, ‘RPN’) [1]. |
| --- | --- |
| Effect category | Type of highest system level effect [2]. |
| Event | Occurrence or change of a particular set of circumstances [3]. |
| Failure | The termination of an item to perform a required function (IEV 191-04-01). |
| Failure cause | Set of circumstances that leads to failure (IEV 192-03-11). |
| Failure effect | Consequence of a failure, within or beyond the boundary of an item (IEV 192-03-08). |
| Failure mode | Manner in which failure occurs (IEV 192-043-17). |
| Fault | The state of an item characterized by inability to perform a required function, excluding the inability during preventive maintenance or other planned actions, or due to lack of external resources (IEV 191-05-01). |

# Abbreviations

| FMEA | Failure modes and effects analysis |
| --- | --- |
| FMES | Failure modes and effects summary |
| FTA | Fault tree analysis |
| RM | Risk matrix |
| RPN | Risk priority number |

# Figures


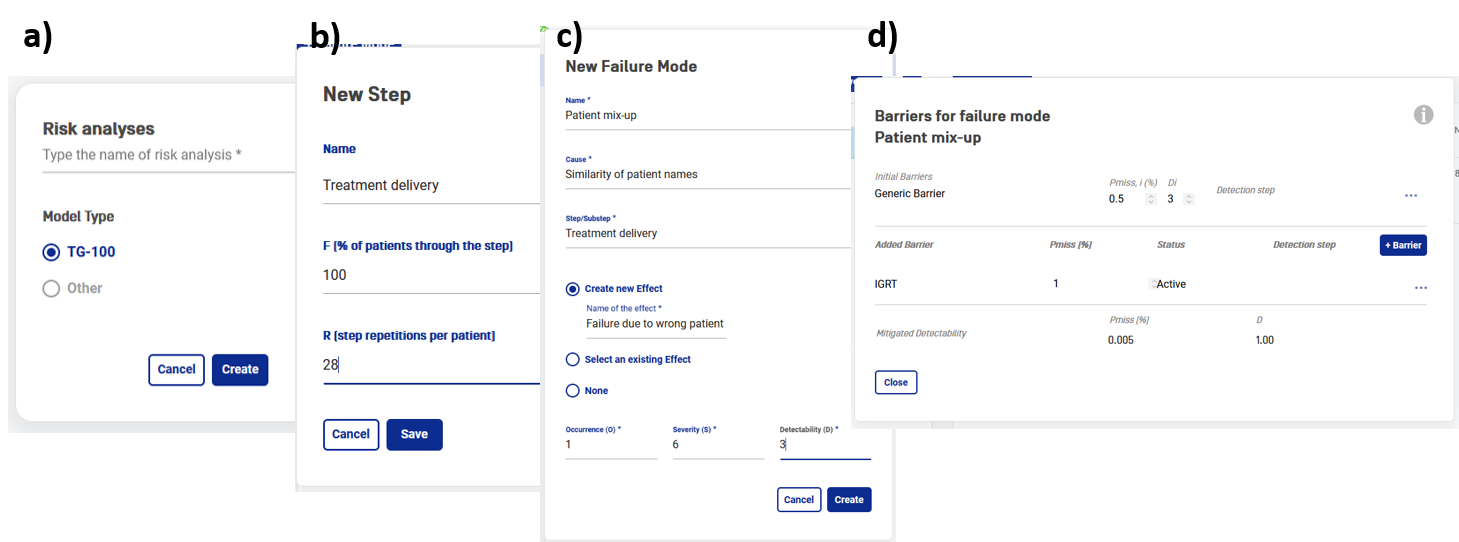


Figure 4: Workflow for the guided risk assessment. a) The user creates the risk assessment, b) then, process steps are added, c) then, failure modes are created and assigned to process steps and d) lastly, barriers are added and assigned to failure modes. Upon adding barriers, optimized RPN evaluations are obtained.


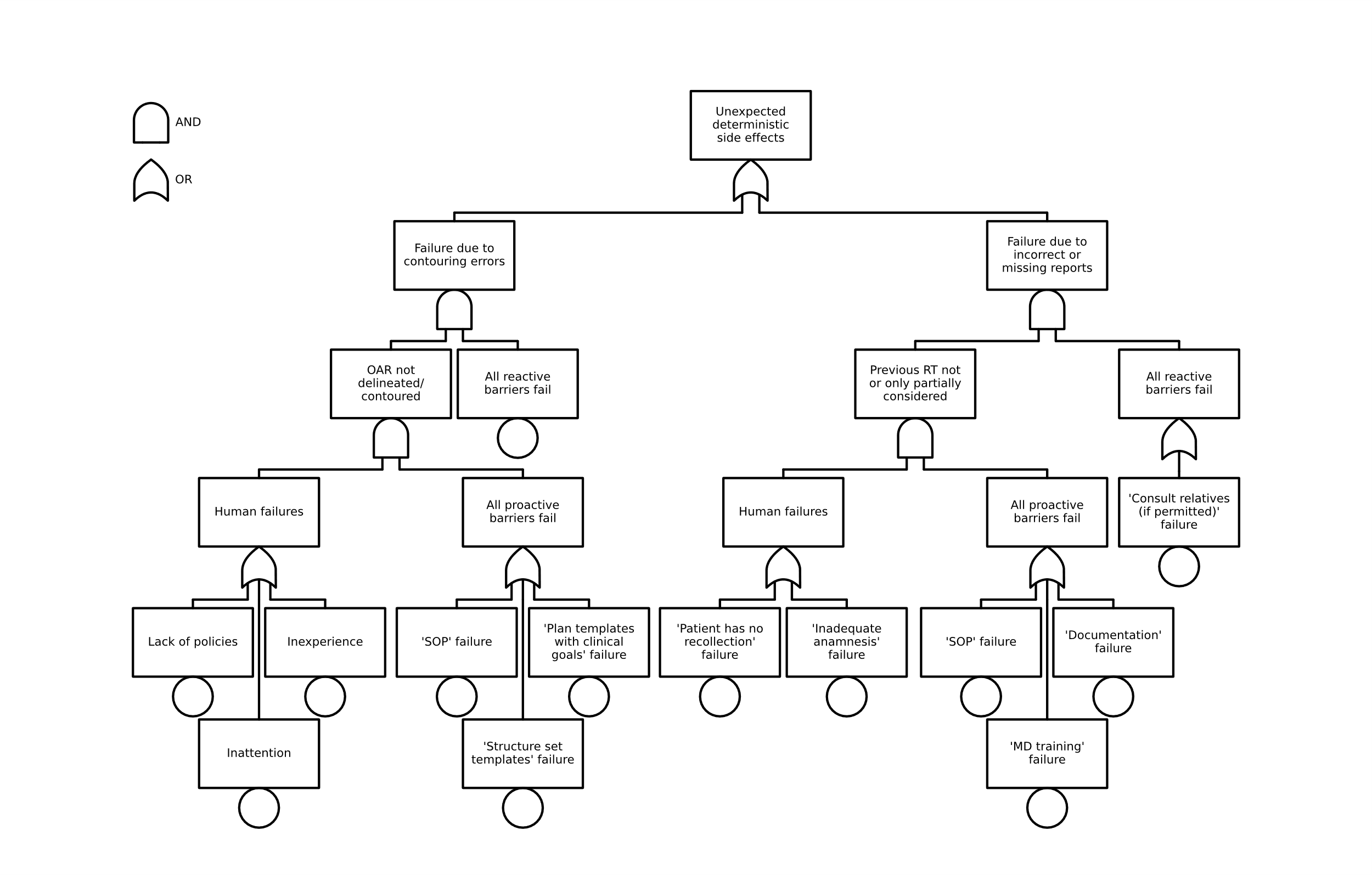


Figure 5: Example of using the results of the failure modes and effects summary to create a fault tree consisting of two FMEA failure modes that potentially cause the same top event.

Remark to Figure 5: A partial fault tree for the two independent failure modes ‘OAR not delineated / contoured’ and ‘previous RT not or only partially considered’ is shown here. These failure modes occur during different processes but could result in the same top event, i. e., ‘unexpected deterministic side effects.’ With the automatically generated fault trees, common events that have previously not been clearly visible on spreadsheets could easier be recognized. Before, these two failure modes had dedicated barriers each, i. e., two standard operating procedures (SOPs) for the first and three for the latter failure mode. A checklist used during initial chart check was identified as a common potential reactive back-up barrier that blocks errors introduced by incorrectly following either of the policies.

# Tables

Table 2 (extended): Scores and corresponding meanings for severity S, occurrence O, detection D and process throughput T taken and modified from [1, 4-8]. Occurrence scores are mapped to probabilities used for calculating reduced occurrence probabilities. The reduced occurrence probability is then mapped back to obtain the improved occurrence score. The same principle also applies to the detection.

| Score | Severity *S* | | Occurrence *O* | Detection *D* | Throughput *T* |
| --- | --- | --- | --- | --- | --- |
|  | Top event | Medical harm | P_occ_ / d^-1^ | P_det_ / % | Patients per year |
| 1 | No effect | No harm | Less than once a year:  < 1/365 | Almost certain—Failure cause or failure mode fully avoided by process due to fail-safe design:  0.01 | 5000 |
| 2 | Inconvenience | Inconvenience  (~ minutes) | Once a year:  1/365 | Very high—Failure cause mechanically detected, propagation of failure prevented (e. g., interlock):  0.2 | 2500 |
| 3 |  | Inconvenience  (~ hours) | Several times a year (~3):  3/365 | High—Process result validated (e. g., trend analysis and prediction, statistical analysis for patient cohort):  0.5 | 1250 |
| 4 | Suboptimal treatment | Side effects  (no intervention) | Once a month:  12/365 | Moderately high—Process result validated (e. g., trend analysis and prediction, statistical analysis for individual patient):  1 | 640 |
| 5 | Unexpected deterministic side effects,  wrong dose,  wrong dose distribution,  wrong location,  wrong treatment period,  wrong volume | Side effects  (intervention) | Several times a month (~3):  36/365 | Moderate—Process result validated (e. g., statistical analysis for individual patient):  2 | 320 |
| 6 |  | Mild toxicity or tumor underdosage | Once a week:  52/365 | Low—Process result verified (e. g., mechanically and automatically, multiple acceptance criteria, 6-eyes principle):  5 | 160 |
| 7 |  | Moderate toxicity or tumor underdosage | Several times a week (~3):  156/365 | Very low—Process result verified (e. g., mechanically and semi-automatically, multiple acceptance criteria, 4-eyes principle):  10 | 80 |
| 8 |  | Severe toxicity or tumor underdosage | Each day:  365/365 | Remote—Process result verified (e. g., manual, random sampling, several acceptance criteria, 2-eyes principle):  20 | 40 |
| 9 |  | Life-threatening | Several times a day (~3):  1095/365 | Very remote—Weak barriers, random sampling:  50 | 20 |
| 10 |  | Premature death | Many times a day:  > 1095/365 | Almost impossible—No barriers implemented:  100 | 10 |

Remark to Table 2: The occurrence and detection probabilities define interval limits. The user may input any numeric probability and the score is then determined by finding the containing interval. For example, if a failure mode is estimated to occur once in ten years, the user would input an occurrence probability of 0.1/365. The occurrence score can then be determined to be 1. As stated in section 2.3, the *a posteriori* RPN is obtained with ${RPN}^{out}=S\cdot O(P_{occ}^{in}\cdot\prod_{i} P_{i,miss}^{proactive})\cdot D(P_{det}^{in}\cdot\prod_{i} P_{i,miss}^{reactive})$. As an example, using the methodology of our department, a failure mode $i$ occurs several times a week (*O* = 7, $P_{occ}^{in}=\frac{156}{365}d^{-1}$). A proactive barrier is expected to reduce the occurrence frequency to 10 % of the initial frequency ($P_{miss}= 0.1$). Therefore, $P_{occ}^{out}=0.1\cdot\frac{156}{365}d^{-1}=\frac{15.6}{365}d^{-1}$ which is slightly more than once a month and hence, the *a posteriori* occurrence score would be *O* = 5. The same principle applies to the detection *D*.

Table 4: Ten highest ranked failure modes for a) normofractionated external beam radiation therapy with existing treatment units (EBRT) and b) with a newly commissioned treatment unit (Halcyon) and residual risk priority numbers after incorporating barriers.

|  |  | FTA | | | FMEA | | | | | | | | | |
| --- | --- | --- | --- | --- | --- | --- | --- | --- | --- | --- | --- | --- | --- | --- |
| Rank by RPN | Rank by RPN* | Top event | Process failure | S | Process step [7] | Failure mode | Failure cause | O | D | T | RPN^in^ | Barriers, proactive | Barriers, reactive | RPN^out^ |
| 1 | 5 | Wrong volume | Failure due to communication errors | 7 | 3.3. Delineation of or­gans-at-risk | Target contour changes after plan approval not communicated (adap­tion to new medi­cal findings) ^a)^ | Lack of communication; Violation of workflow procedure | 7 | 6 | 3 | 294 | SOP; Locking plan and structure data | Check of contours and approval status by attending RO | 140 |
| 2 | 1 | Wrong dose | Failure due to erroneous measurement | 8 | 8.4. Ongoing quality management (e. g., daily, monthly, annual QA, etc.) | Wrong placement of measuring instruments ^b)^ | Lack of optical or mechanical distance indicators in beam isocenter | 6 | 6 | 5 | 288 | Usage of vendor workflow (‘follow the blue’) | Image-guided check (MV @ 0° and 90° gantry angle) | 32 |
| 3 | 7 | Unexpected deterministic side effects | Failure due to contouring errors | 8 | 3.3. Delineation of or­gans-at-risk | OAR not delineated/contoured ^a)^ | Lack of policies; Inatten­tion; Inexperi­ence | 7 | 5 | 2 | 280 | SOP; Structure set templates; plan templates with clinical goals | - | 120 |
| 4 | 2 | Wrong dose | Failure due to erroneous measurement | 10 | 8.2. Commissioning | Inaccurate beam model in non-vendor TPS ^b)^ | Wrong usage of non-vendor TPS; Wrong data transfer of beam data; Erroneous measurement of beam data | 7 | 4 | 5 | 280 | Measurements according to vendor guide | Commissioning of non-vendor TPS and comparison with vendor TPS after validation of beam data in vendor TPS | 40 |
| 5 | 3 | Wrong dose | Failure due to organizational issues | 6 | 7.2. Final chart check | Documentation in ARIA and MOSAIQ inconsistent (Rx, RT protocol, Tx courses) ^b)^ | Data entry/transfer error, Lack of synchronization | 7 | 5 | 5 | 210 | Archiving ARIA PDF report in MOSAIQ upon final fraction | - | 60 |
| 6 | 4 | Inconvenience | Failure due to delay | 2 | 5.4. Selection of intended course/session | Treatment plan not deliverable ^b)^ | Invalid plan–Only plans coming from vendor TPS may be delivered | 10 | 10 | 5 | 200 | Instruction of correct export of plan from non-vendor TPS to vendor TPS to treatment unit | Scripted plausibility check within TPS | 20 |
| 7 | 8 | Unexpected deterministic side effects | Failure due to incorrect or missing reports | 8 | 1.8. Evaluation of previ­ous radio­therapy treatments | Previous RT not or only partially considered ^a)^ | Patient has no recollection; Inadequate anamnesis | 3 | 7 | 3 | 168 | SOP; MD training; documentation | Consult relatives (if permitted) | 80 |
| 8 | 9 | Non-radiation re­lated injury | Failure due to poor execution | 9 | 2.7. Contrast administra­tion | Inadequate handling of anaphylac­tic shock ^a)^ | Lack of experience; Lack of training | 2 | 9 | 3 | 162 | SOP; Training | Regular check of close-at-hand access and completeness of emergency drugs | 81 |
| 9 | 14 | Wrong treatment period | Failure due to organizational issues | 9 | 6.6. Weekly physician management visit, social work, nutrition and nursing | Discontinuation of treatment of patients with COVID-19 ^a)^ | Lack of preventive measures and procedures to protect staff | 8 | 2 | 2 | 144 | SOP; Safety equipment; Vaccination | - | 18 |
| 10 | 16 | Inconvenience | Failure due to delay | 7 | 1.12. Selection of clini­cal protocol | Wrong protocol ^a)^ | Insufficient information during time of treatment decision | 5 | 4 | 2 | 140 | Revise patient consent before treat­ment, including plan and new find­ings or changes | - | 84 |

# References

[1] *IEC 60812:2018. Failure modes and effects analysis (FMEA and FMECA)*, 2018.

[2] Kritzinger D, *Aircraft system safety : assessments for initial airworthiness certification*. Duxford: Woodhead Pub. (in English), 2017.

[3] *ISO Guide 73:2009. Risk management — Vocabulary*, 2009.

[4] Huq MS, Fraass BA, Dunscombe PB, Gibbons JP, Jr., Ibbott GS, Mundt AJ *et al.* The report of Task Group 100 of the AAPM: Application of risk analysis methods to radiation therapy quality management, *Med Phys,* vol. 43, no. 7, p. 4209, Jul 2016, doi: 10.1118/1.4947547.

[5] Janssens PMW and van der Horst A. Improved prospective risk analysis for clinical laboratories compensated for the throughput in processes, *Clin Chem Lab Med,* vol. 56, no. 11, pp. 1878-85, Oct 25 2018, doi: 10.1515/cclm-2018-0109.

[6] *Verordnung zum Schutz vor der schädlichen Wirkung ionisierender Strahlung,* Teil I Nr. 41, 2018.

[7] Ford EC, Fong de Los Santos L, Pawlicki T, Sutlief S, and Dunscombe P. Consensus recommendations for incident learning database structures in radiation oncology, *Med Phys,* vol. 39, no. 12, pp. 7272-90, Dec 2012, doi: 10.1118/1.4764914.

[8] *Common Terminology Criteria for Adverse Events (CTCAE) Version 5.* US Department of Health and Human Services, 2017.
